# Grape seed proanthocyanidin extract protects human lens epithelial cells from oxidative stress via reducing NF-кB and MAPK protein expression

**Published:** 2011-01-20

**Authors:** Zhiyan Jia, Zhen Song, Yuhui Zhao, Xiurong Wang, Ping Liu

**Affiliations:** 1 Department of Ophthalmology, The First Affiliated Hospital of Harbin Medical University, Harbin, P.R. China; 2 Animal Influenza Laboratory of the Ministry of Agriculture and National Key Laboratory of Veterinary Biotechnology, Harbin Veterinary Research Institute, Chinese Academy of Agricultural Sciences, Harbin, P.R. China

## Abstract

**Purpose:**

Oxidative damage induced by H_2_O_2_ treatment can irreversibly damage the lens epithelium, resulting in cell death and cataract. Grape seed extract (GSE) is a widely consumed dietary supplement that has the capability to scavenge oxidants and free radicals. GSE contain 70%–95% standardized proanthocyanidins. The study described herein investigated the protective effect of Grape seed proanthocyanidin extract (GSPE) on H_2_O_2_-induced oxidative stress in human lens epithelial B-3 (HLEB-3) cells and the possible molecular mechanism involved.

**Methods:**

HLE-B3 cells exposed to different doses of H_2_O_2_ were cultured with various concentrations of GSPE and subsequently monitored for cell viability by the 4,5-dimethylthiazol-2-yl-2,5-diphenyltetrazolium bromide (MTT) assay. The apoptosis rate and ROS generation were detected by flow cytometric analysis. Expression of NF-кB/P65 and mitogen activated protein kinase (MAPK) proteins were measured by western blot.

**Results:**

GSPE clearly reduced H_2_O_2_ induced cell apoptosis and reactive oxygen species (ROS) generation and protected HLEB-3 cells from H_2_O_2_ induced oxidative damage. GSPE depressed H_2_O_2_-induced activation and translocation of NF-кB/p65. GSPE also depressed H_2_O_2_-induced phosphorylation of the p38 and c-Jun N-terminal kinase (JNK) proteins of the MAPK family at various time points studied.

**Conclusions:**

GSPE could be useful in attenuation of H_2_O_2_-induced oxidative stress and the activation of NF-кB and MAPK signaling in HLE-B3 cells, which suggests that GSPE has a potential protective effect against cataractogenesis.

## Introduction

Cataract remains the leading cause of visual disability and blindness worldwide. At present, the only remedy is surgical removal of the cataractous lens and substituting it with a lens made of synthetic polymers. However, the incidence is so large that the available surgical facilities are unable to cope up with the problem. In addition to these, postoperative complications can occur such as posterior capsular opacification, endophthalmitis, and uncorrected residual refractive error. Therefore, it is so necessary to search for pharmacological intervention that will maintain the transparency of the lens.

Loss of transparency during human cataract formation results from a variety of complex metabolic and physiologic mechanisms. In the cells, reactive oxygen species (ROS) may initiate a surge of toxic biochemical reactions such as peroxidation of membrane lipids and extensive damage to proteins causing intracellular protein aggregation and precipitation and eventually leading to lens opacification [1,2]. Several studies have shown that exposure of lens epithelial cells to H_2_O_2_ increases ROS production and oxidative stress [3,4].

Proanthocyanidins are natural compounds found in high concentrations in fruits, vegetables, wine, tea, nuts, and seeds [5,6]. They are a class of phenolic compounds that take the form of oligomers or polymers of polyhydroxy flavan-3-ol units, such as (+)-catechin and (−)-epicatechin. The seeds of the grape are particularly rich source of proanthocyanidins. The grape seed proanthocyanidins extract (GSPE) are mainly dimers, trimers, and highly polymerized oligomers of monomeric catechins [7,8]. Proanthocyanidins possess a wide array of pharmacological and biochemical actions including anti-inflammatory, anti-carcinogenic activity, and cardioprotective biologic effects [5,6,9,10]. In addition to these, GSPE have been shown to be potent antioxidants and free radical scavengers, being more effective than either ascorbic acid or vitamin E [5,9]. In vivo experiments, it has been reported that GSPE can prevent cataract formation in rats predisposed to hereditary cataracts [11]. Besides, some experiments have given evidences that GSPE can prevent selenite cataract development in rats [12,13]. GSPE is marketed as a dietary supplement in the United States, due to their powerful antioxidant activity, low toxicity and no genotoxic potential [14]. However, until now, there is no study about the effect of GSPE on H_2_O_2_-induced oxidative stress and the precise mechanism of signal transduction in human lens epithelial (HLE) cells.

Dietary modulation of the mitogen-activated protein kinases (MAP K) pathway and nuclear factor kappa-B (NF-кB) has emerged as a potential target of dietary antioxidants. Members of the two pathways, as well as protein38 (p38), c-jun N-terminal kinase (JNK), and NF-кB/protein65 (p65), are involved in the regulation of cellular differentiation, migration, proliferation and survival [15,16].

Based on these recent studies, we hypothesized that GSPE would protect HLE cells from oxidative stress by influencing several signaling pathways and thus would be beneficial in the treatment of cataract. The study was designed to determine whether GSPE could reduce H_2_O_2_-induced cell apoptosis and cell death in cultured HLEB-3 cells. We also detected if GSPE can scavenge ROS accumulation. At last, we investigated the mechanism of GSPE in protecting the HLEB-3 cells from oxidative damages.

## Methods

### Materials

GSPE, constituting of 95% (wt/wt) proanthocyanidins, was provided by JF-NATURAL Corp. (Tian Jin, China) and was dissolved in deionized water. The same lot of GSPE was used for all experiments. HLE-B3 cells, a human lens epithelial cell line immortalized by SV-40 viral transformation [17], were purchased from the ATCC (American Type Culture Collection; Rockville, MD). Fetal bovine serum (FBS) and Dulbecco’s modified Eagle’s medium (DMEM) were obtained from Gibco (Grand Island, NY). Anti-JNK, anti-phosphorylation of JNK (p-JNK), anti-p38 and anti-phosphorylation of p38 (p-p38) were purchased from Santa Cruz Biotechnology Inc. (Santa Cruz, CA).

### Cell culture

HLE-B3 cells were maintained at 37 °C in DMEM supplemented with 20% FBS and 1% antimicrobial solution (Sigma Chemical Co., St. Louis, MO) in an environment composed of 5% CO_2_/95% O_2_. When grown to 75%–80% confluence, the cells were treated with the indicated concentration of H_2_O_2_ for the required time or pretreated with GSPE for different time before the H_2_O_2_ treatment. At the indicated time points, the cells were collected for different assays.

### Measurement of cell viability

The 4,5-dimethylthiazol-2-yl-2,5-diphenyltetrazolium bromide (MTT) assay was used to verify the viability of HLEB-3 cells. Cells were plated at a density of 1×10^4^ cells/well in 96-well microplates. After a 24 h incubation, cells were pretreated with 2.5, 5.0, 10.0, and 20.0 mg/l GSPE for 12 h, 24 h, and 48 h. After incubation for the indicated time, cells were treated with 50, 100, and 200 μmol/l H_2_O_2_ in combination for 24 h. Cells were incubated with 20 μl of MTT solution (0.5 mg/ml) for 4 h at 37 °C, and the solution was then replaced with 200 μl DMSO after the incubation. The absorbance was measured at 490 nm by a microplate reader (ELx800; BioTek, Winooski, VT).

### Apoptosis assay

For the quantification of apoptotic death, Cells were grown on a six-well plate at 1×10^5^ cells per plate and pretreated with or without different concentration of GSPE for 12 h before 100 μmol/l H_2_O_2_ treatment for 24 h. Thereafter, cells were collected and stained with Annexin V and PI using Vybrant Apoptosis Assay Kit 2 (Molecular Probes, Eugene, OR) essentially following the instructions of the manufacturer. Stained cells were analyzed using Cell-Quest software.

### ROS detection

The production of ROS was monitored using flow cytometry. ROS production was quantified by the 2',7'-dichlorofluorescin-diacetate (DCFH-DA) method based on the ROS-dependent oxidation of DCFH-DA to DCF. Cells were incubated with 10.0 μM DCFH-DA (Molecular Probes, Eugene, OR) for 30 min. They were then washed and incubated with complete medium for 2 h. ROS generation was determined using FAC Scan flow cytometry and results were analyzed using Cell-Quest software (Becton, Dickinson and Company, Franklin Lakes, NJ).

### Western blotting

After treatments, cultured cells were washed with cold PBS and then lysed in a buffer containing 50 mM Tris-HCl, pH 7.5, 150 mM NaCl, 1 mM Na_2_EDTA, 1 mM EDTA, 1% Triton-X100, 2.5 mM sodium pyrophosphate, 1 mM β-glycerophosphate, 1 mM Na_3_VO_4_, 1 mM NaF, 1 μg/ml leupeptin, and 1 mM phenylmethanesulfonyl fluoride (PMSF). Nuclear extracts were collected according to the instruction of the nuclear extract kit (Active Motif, Carlsbad, CA). Cell lysates were collected and their protein concentration was evaluated using a protein assay (Bio-Rad, Melville, NY). After boiling at 100 °C for 5 min, the proteins were resolved by electrophoresis on 10%–15% SDS-polyacrylamide gels (20 μg proteins per lane) and transferred to a polyvinylidene difluoride (PVDF) membrane (Millipore, Milford, MA). Membranes was incubated in 5% fat-free milk powder in Tris-buffered saline solution–Tween-20 (TBST) for 1 h at room temperature and then exposed to the primary antibodies at 4 °C overnight. After rinsing in TBST (3 times for 5 min each time), the membranes were incubated with HRP-conjugated secondary antibodies for 1 h at room temperature. Membranes were then detected by enhanced chemiluminescence (ECL) western detection reagents (Amersham, Pittsburgh, PA).

### Statistical analysis

All experiments were performed at least three times and the results presented are from representative experiments. Data were compared by one-way ANOVA. For all experiments, data were reported as mean±SD as indicated, and p values <0.05 were considered significant.

## Results

### GSPE increases the viability of HLEB-3 cells treated by H_2_O_2_

**Figure 1:**
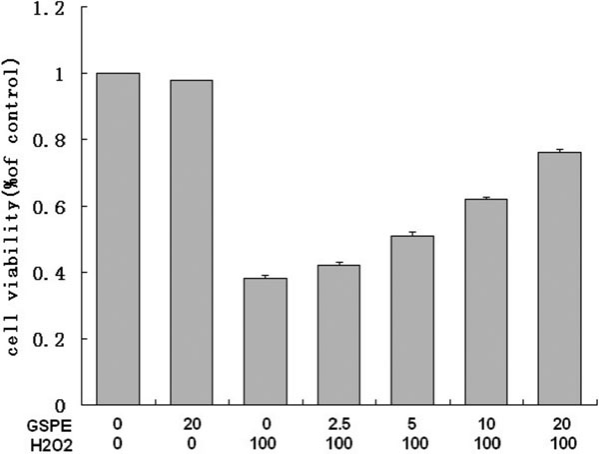
GAPE increased the viability of cells in concentration-dependent manner. The cell viability of HLEB-3 cells pretreated with GSPE at different concentrations (0, 2.5, 5.0, 10.0, and 20.0 mg/l) for 12 h before H_2_O_2_ (100 μmol/l) treatment for 24 h was estimated by using MTT. The results showed a concentration- dependent increase of GSPE in the viability of cells.

**Figure 2:**
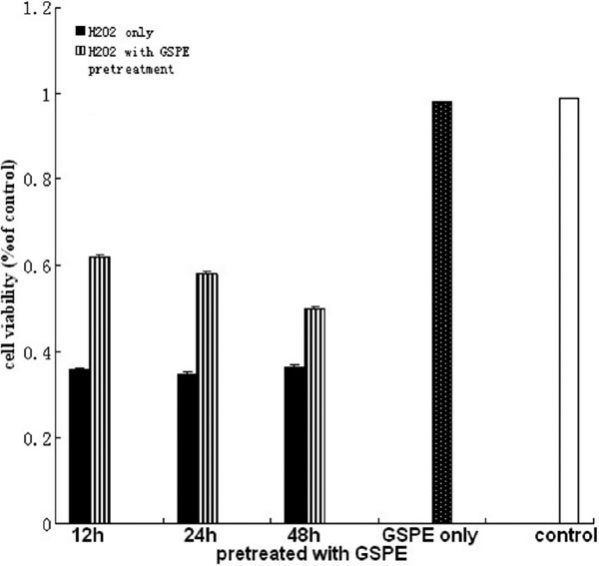
The pretreatment with GSPE for 12 h was the optimal time. The cells were incubated with 20.0 mg/l GSPE for different time (12, 24, 48 h), then treated with 100 μmol/l H_2_O_2_ for 24 h. The cell viability was measured by MTT assay. The results showed that pretreatment with GSPE for 12 h was the optimal time.

**Figure 3:**
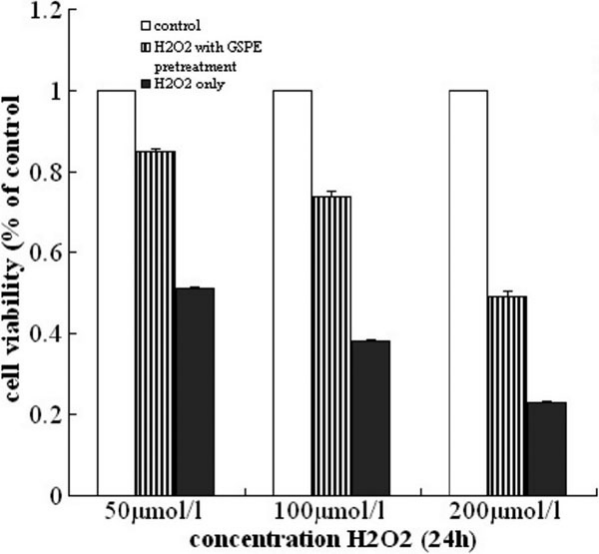
The protecting effect of GSPE was inverse ratio to H_2_O_2_ concentration. The cells were pretreated with 20.0 mg/l GSPE for 12 h, then exposed to different concentration of H_2_O_2_ (50, 100, and 200 μmol/l) for 24 h. The cell viability was measured by MTT assay. The results showed the protecting effect of GSPE was inverse ratio to H_2_O_2_ concentration.

First we studied the effect of GSPE on the viability of HLEB-3 cells treated by H_2_O_2._ After HLEB-3 cells were treated with GSPE at the concentrations ranging from 0 to 20 mg/l for 12 h, these HLEB-3 cells were incubated with 100 μmol/l H_2_O_2_ for 24 h. The results showed a concentration- dependent increase in the viability of cells measured in terms of absorbance of color formed by reduction of MTT dye by live cells (Figure 1).Thus 20 mg/l was determined as the optimal dose for GSPE pretreatment. Then we investigated whether the protective effect of GSPE vary with incubation time of GSPE. Increase in treatment times to 24 and 48 h reduced the viability. Figure 2 showed that pretreatment with GSPE for 12 h was the optimal time compared with others. Finally, we selected a 12 h pretreatment with 20 mg/l GSPE before exposure to different concentrations of H_2_O_2_ and found that the protecting effect of GSPE was inverse ratio to H_2_O_2_ concentration, as seen in Figure 3. From these results, it could be concluded that GSPE had a protective role against H_2_O_2_ induced cell damage and the optimal incubation time for GSPE pretreatment is 12 h.

### GSPE reduced apoptotic death in HLEB-3 cells treated by H_2_O_2_

**Figure 4:**
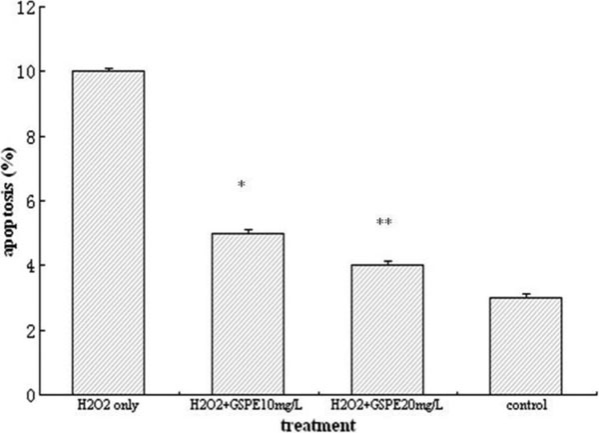
GSPE significantly decreased the apoptosis rate of HLEB-3 cells. The HLEB-3 cells were incubated with 10 mg/l and 20 mg/l GSPE for 12 h then treated with 100 μmol/l H_2_O_2_ for 24 h. Flow cytometric analysis of annexin V-PI stained cells showed GSPE significantly decreased the apoptosis rate of HLEB-3 cells. **p<0.01; *p<0.05.

Since we observed that GSPE treatment causes significant protection in HLEB-3 cells, we next examined if the protective effects induced by GSPE involve reduction of apoptosis. Based on the results from MTT assay, the HLEB-3 cells were incubated with 10 mg/l and 20 mg/l GSPE for 12 h then treated with 100 μmol/l H_2_O_2_ for 24 h. Flow cytometric analysis of annexin V-PI stained cells revealed that indeed GSPE clearly decreased H_2_O_2_ induced apoptosis. As shown in Figure 4 the results were displayed as histograms.

### GSPE reduces the generation of ROS induced by H_2_O_2_ in HLEB-3 cells

**Figure 5:**
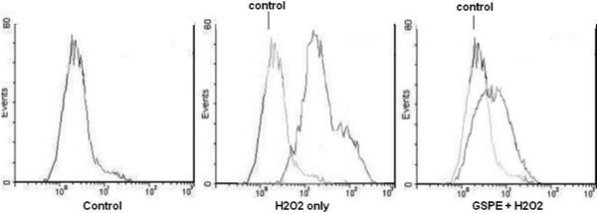
Effect of GSPE on H_2_O_2_-induced generation of reactive oxygen species in HLEB-3 cells. HLEB-3 cells were pretreated with 20 mg/l GSPE for 12 h followed by treatment with 100 μmol/l H_2_O_2_. The ROS production was measured by DCF flow cytometry as described in Methods. The result showed GSPE pretreatment reduced ROS generation induced by H_2_O_2_.

DCF-DA was used to carry out the generation of ROS. As shown in Figure 5, treatment with 100 μmol/l H_2_O_2_ significantly enhanced the generation of ROS. Pretreatment with 20 mg/l GSPE distinctly reduced the generation of ROS. Three repeated experiments showed GSPE pretreatment inhibited ROS generation induced by H_2_O_2_, which had statistical significance.

### GSPE reduces H_2_O_2_-induced activation and translocation of NF-кB in HLEB-3 cells

**Figure 6:**
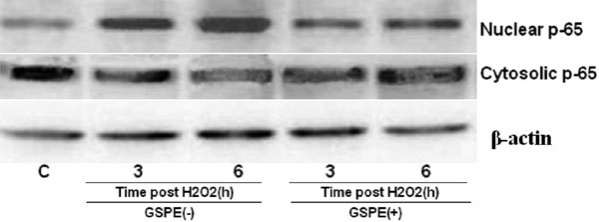
Effect of GSPE on H_2_O_2_-induced activation of NF-кB in HLEB-3 cells. LEB-3 cells were incubated with 20 mg/l GSPE for 12 h. Cells were harvested at 3 h and 6 h time points after treated with 100 μmol/l H_2_O_2_. Western blot indicated that treatment with GSPE before H_2_O_2_ markedly decreased H_2_O_2_-induced activation of NF-кB/p65.

Next, we examined protein level of transcription factor—NF-κB. HLEB-3 cells were incubated with 20 mg/l GSPE for 12 h. Cells were harvested at 3 h and 6 h time points after treated with 100 μmol/l H_2_O_2_, and cell lysates were prepared to determine the activation of NF-кB . Western blotting indicated that treatment with GSPE before H_2_O_2_ markedly abrogated H_2_O_2_-induced activation of NF-кB/p65 compared with control cells. Activation of NF-кB was based on the detection of its translocation into cell nuclei from its initial location in the cytoplasm where it exists in an inactive form. Cells treated by H_2_O_2_ exhibited an enhancement of nuclear NF-кB/p65 and a reduction of cytosolic NF-кB /p65 at 3h, and this became more evident at 6 h (Figure 6). Western blot indicated that treatment with GSPE before H_2_O_2_ markedly abrogated H_2_O_2_-induced activation of NF-кB/p65.

### GSPE reduces H_2_O_2_-induced phosphorylation of the MAPK pathway

**Figure 7:**
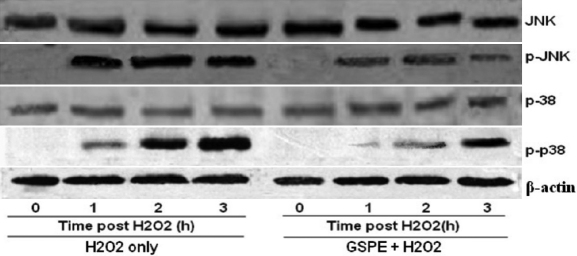
Effect of GSPE on H_2_O_2_-induced phosphorylation of the MAPK pathway. HLEB-3 cells were incubated with 20 mg/l GSPE for 12 h. Cells were harvested at 1, 2, and 3 h time points after treated with 100 μmol/l H_2_O_2_, and p38 and JNK activities were evaluated by western blotting. GSPE markedly reduced H_2_O_2_induced phosphorylation of p38 and JNK at each time point studied. The total amount of JNK and p38 remained unchanged at each time point studied.

Whole cell extracts were prepared and analyzed using antibodies against the active phosphorylated forms of JNK and p38. As shown in Figure 7, phosphorylation of p38 and JNK started 1 h after H_2_O_2_ treatment. Western blotting and subsequent measurement of the intensity of the bands relative to the total amount of p38 and JNK phosphorylation indicated that treatment with GSPE markedly depressed H_2_O_2_ induced phosphorylation of p38 and JNK at each time point studied. Further, the total amount of JNK and p38 remained unchanged at each time point studied. These results suggested that GSPE pretreatment suppressed JNK and p38 phosphorylation.

## Discussion

Cataract formation is associated with cell signaling, cell migration, and inflammation [18,19]. It has been found that GSPE attenuates these processes and finally cause slowing or inhibition of cataract formation [11-13]. Furthermore, GSPE has been found to be nontoxic in other studies [14]. Therefore, the study reported here was to evaluate the effects of GSPE on factors that contribute to cataract formation, including ROS production and cell signaling. The results suggest that GSPE is a potent inhibitor of H_2_O_2_ -induced oxidative stress and the activation of NF-кB and MAPK signaling in human lens epithelial cells. Thus it has a potential therapeutic role in the prevention of cataract. Our experiment is the first to show GSPE protective function in HLE cells and its mechanism against oxidative stress.

In our experiment, H_2_O_2_ was used as the oxidant model of classical oxidative stress. H_2_O_2_ treated cells showed an increased production of intracellular ROS and a high apoptosis rate. However GSPE pretreatment can significantly depress the ROS production in H_2_O_2_ treated HLEB-3 cells, which suggested a primary role of GSPE in protection against ROS. Moreover, flow cytometry analysis using PI and annexin V showed that H_2_O_2_-induced cell apoptosis in HLEB-3 cells were significantly reduced by GSPE pretreatment. These results indicated that GSPE can not only reduce the production of ROS, but also prevent HLEB-3 cells from apoptosis caused by H_2_O_2_.

To investigate the protective effect of GSPE against oxidative stress in HLEB-3 cells, we looked into the potential pathway involved. NF-кB is one of the most ubiquitous transcription factors. The genes regulated by NF-κB include apoptosis, cell adhesion, proliferation, inflammation, and cellular-stress response [20,21]. In unstimulated cells, NF-кB resides in the cytoplasm in an inactive complex with inhibitor kappa B. Pathogenic stimuli induce phosphorylation and the subsequent release of inhibitor kappa B, resulting in NF-кB translocation to the nucleus where it binds to DNA control elements and thus influences the transcription of certain specific genes [22,23]. Many studies indicate that NF-κB is a stress sensitive transcription factor and its activation is regulated by reactive oxygen species. In our study, NF-кB is activated by H_2_O_2_ and subsequently translocated into the nucleus, which is consistent with these studies [24,25]. However, the activation and translocation were effectively decreased by pretreatment with GSPE. Therefore, this observation provides a possible mechanism for the effect of GSPE.

The MAPK signaling pathway is an important upstream regulator of transcriptional factor activities and their signaling affects a wide variety of extracellular stimuli into intracellular events and thus control the activities of downstream transcription factors [26,27]. Several investigations reported that GSPE reduced the oxidative stress of various cells via inhibition of the MAPK signaling cascade [28,29]. It is generally accepted that ERK activation is essential for cell survival, whereas activation of JNK and p38 is thought to play an important role in cell death. Therefore, we investigated whether the p38 and JNK pathways were inhibited by treatment with GSPE. Our data showed that H_2_O_2_-induced p38 and JNK activation was significantly reduced when cells were pretreated with GSPE. These results suggested that GSPE reduced H_2_O_2_-induced intracellular ROS accumulation, decreased the intracellular oxidative level, indirectly prevented HLEB-3 cells from p38 and JNK signaling pathway, and further proved that GSPE plays an important role in protecting HLEB-3 cells against oxidative stress.

Though the exact mechanism of the reduction of H_2_O_2_-induced phosphorylation of MAPK proteins by GSPE is not clear based on the present data, previous studies have shown proteins of the MAPK family to be involved in the activation of NF-кB [30-32]. In this experiment, it appears that the antioxidant property of GSPE contributed to the reduction of the H_2_O_2_-induced phosphorylation of MAPKs through both a modulation of ROS and prevention of downstream events such as NF-кB activation. Therefore, the reduction of the MAPK and NF-кB signaling pathways could potentially be used by GSPE to activate certain antioxidant-responsive genes to protect against the H_2_O_2_-induced oxidative stress in HLE cells.

Some researches have demonstrated that the contributions of the various receptors in human lens epithelial cells differ in primary and immortalized cells [33]. This is may be one of reasons why our results are slightly different from those of experiments in which primary cells were used.

In conclusion, GSPE protects HLE cells from H_2_O_2_-induced oxidative stress by reducing the generation of ROS and modulating the activation of NF-кB and MAPK pathways. GSPE possesses a potential pharmacological application in attenuating H_2_O_2_-induced oxidative stress, suggesting a protective effect against cataractogenesis. On the basis of the data reported here, there is strong evidence that GSPE could potentially protect HLEB-3 cells from the damaging effects of oxidative stress. Delaying cataract formation would be beneficial because it has been estimated that a delay of 10 years for cataract formation in humans would decrease sight-threatening cataracts by 45% [34]. Therefore, even a slight delay in the progression of cataract formation could provide increases in visual acuity.
